# *Blastocystis*: how do specific diets and human gut microbiota affect its development and pathogenicity?

**DOI:** 10.1007/s10096-017-2965-0

**Published:** 2017-03-22

**Authors:** M. Lepczyńska, J. Białkowska, E. Dzika, K. Piskorz-Ogórek, J. Korycińska

**Affiliations:** 1 Department of Medical Biology, Faculty of Medical Sciences, University of Warmia and Mazury, Żołnierska 14 C, Olsztyn, 10-561 Poland; 2 Department of Neurology and Neurosurgery, Faculty of Medical Sciences, University of Warmia and Mazury, Warszawska 30, Olsztyn, Poland; 3 Department of Nursing, Faculty of Medical Sciences, University of Warmia and Mazury, Żołnierska 14 C, Olsztyn, Poland; 4 Regional Specialized Children’s Hospital in Olsztyn, Żołnierska 18A, Olsztyn, Poland

## Abstract

*Blastocystis* is an enteric parasite that inhabits the gastrointestinal tract of humans and many animals. This emerging parasite has a worldwide distribution. It is often identified as the most common eukaryotic organism reported in human fecal samples. This parasite is recognized and diagnosed more often than ever before. Furthermore, some strains develop resistance against currently recommended drugs, such as metronidazole; therefore, the use of natural remedies or special diets has many positive aspects that may address this problem. The goal of this review is to compare natural treatments and various diets against the efficacy of drugs, and describe their influence on the composition of the gut microbiota, which affects *Blastocystis* growth and the occurrence of symptoms. This article reviews important work in the literature, including the classification, life cycle, epidemiology, pathogenesis, pathogenicity, genetics, biology, and treatment of *Blastocystis*. It also includes a review of the current knowledge about human gut microbiota and various diets proposed for *Blastocystis* eradication. The literature has revealed that garlic, ginger, some medical plants, and many spices contain the most effective organic compounds for parasite eradication. They work by inhibiting parasitic enzymes and nucleic acids, as well as by inhibiting protein synthesis. The efficacy of any specific organic compound depends on the *Blastocystis* subtype, and, consequently, on its immunity to treatment. In conclusion, the article discusses the findings that human gut microbiota composition triggers important mechanisms at the molecular level, and, thus, has a crucial influence on the parasitic pathogenicity.

## Meet *Blastocystis* sp.: classification and life cycle

*Blastocystis* is a unicellular protist present throughout the world in the intestines of both healthy and symptomatic humans and animals [1]. Its pathogenic potential is still controversial [2–5]. For many years, it has been suggested that *Blastocystis* is a commensal organism living in the human intestine. Originally, the parasite was considered to be a harmless yeast until the 1970s, when evidence showed that *Blastocystis* was actually a protist [6], belonging to the Stramenopiles line of eukaryotic organisms. In 2009, Irikov et al. [7] proposed to place this organism in a separate sixth kingdom named “Chromista”. Until recently, the taxonomy of *Blastocystis* was based on the host from which it was isolated (*B. hominis* from humans, *B. ratti* from rats, etc.). Modern phylogenic studies have identified a lack of host specificity for *B. hominis* and *B. ratti*, and, therefore, have proposed to summarize the name as “*Blastocystis* species”, with different ribosomal lineages classified into specific subtypes [8, 9].

Morphologically indistinguishable phylogenetic inferences from small subunit rDNA (SSU rRNA) gene sequence analyses revealed considerable genetic divergence among *Blastocystis* isolated from humans and animals, with a total of 17 subtypes being identified so far [10]. The majority of human *Blastocystis* carriage is attributable to ST3 [11, 12], appearing quite rarely in non-primate hosts, suggesting that ST3 may be the only subtype (ST) of human/primate origin [13]. Several STs of supposed animal origin are zoonotic, with the ability to infect humans at different frequencies. Therefore, a higher risk of *Blastocystis* infection has been shown in people living in rural areas and/or with close animal contact [14]. Phylogenetic trees comparing *Blastocystis* SSU rDNA sequences have been developed by many researchers in many countries [11, 15, 16]. The trees are mostly created using the neighbor-joining method (with the maximum composite likelihood model) based on either complete SSU rRNA genes or the hypervariable region at the 5′-end of the SSU rRNA gene. Relationships among *Blastocystis* subtypes occurring in humans have been analyzed and groups of STs were named as clades. One clade consists of STs 1, 2, and 5 (the closest relation between ST1 and ST2); another clade consists of STs 3, 4, and 8 (the closest relation between ST3 and ST8). A third clade consists of STs 6, 7, and 9 (where the closest relation is between STs 6 and 9) [8, 11].

The parasite has been known since the early 1900s [1], but only in the last decade has the biology and pathogenicity of this parasite undergone more intensive studies. However, the lack of standardization in detection techniques and methods for molecular characterization has led to confusion and misinterpretation of the data. A number of studies suggested a linkage between *Blastocystis* and gastrointestinal disorders, skin symptoms, and many other problems. Recent epidemiological data demonstrate the association of *Blastocystis* with a variety of disorders [1, 17], such as diarrhea, abdominal pain, fatigue, constipation, flatulence, chronic gastrointestinal illnesses (irritable bowel syndrome, IBS), and skin rash or urticaria [18, 19]. *Blastocystis* has been found in both patients with gastrointestinal symptoms and asymptomatic individuals [20, 21]. According to a number of studies, the life cycle of *Blastocystis* and its pathogenic aspects are still unclear.

*Blastocystis* infects at least 5–15% of individuals in developed countries and 50–100% of individuals in developing countries [22, 23]. The difference can be partly explained by poor hygiene practices and consumption of contaminated water or food in developing countries [24]. The fecal–oral route is considered to be the main mode of transmission. Controversy regarding the commensal or pathogenic nature of the infection has not changed for decades. Many case reports and epidemiological and microbiological studies support a pathogenic role of *Blastocystis* in causing intestinal inflammation and urticarial symptoms [25], while there are many reports on asymptomatic colonization by *Blastocystis* [26, 27]. Other aspects, including mode of transmission, pathogenicity, life cycle, and molecular biology, remain largely unclear.

The prevalence of *Blastocystis* infection is higher than that of other intestinal parasites, such as *Giardia*, *Entamoeba*, or *Cryptosporidium* [4]. In immunocompromised individuals, such as those with human immunodeficiency virus (HIV) infection, the prevalence of *Blastocystis* is between 30 and 38% in developed countries [28, 29]. It is suggested that *Blastocystis* is linked with diarrhea in immunocompromised hosts, such as HIV-infected persons, and nutrition status may be one of the important risk factors associated with co-infections [3]. Children and the elderly appear to be highly susceptible to *Blastocystis* infection [30, 31], while other researchers have suggested that people between 30 and 50 years of age are most prone to being infected by *Blastocystis* [32–34].

Six different morphological forms of the parasite have been reported (vacuolar, granular, amoeboid, avacuolar, multivacuolar, and cystic), with the cyst being the infective stage and the amoeboid form supposedly playing a more active role in the development of clinical manifestations [35]. The vacuolar form is most commonly observed in both laboratory culture and stool samples [36, 37].

The life cycle and transmission of the parasite are still under intensive investigation. Two types of reproduction have been described: asexual reproduction by binary fission and sexual reproduction by autogamy to form a primary cyst [3, 38], which is the only transmissible form of *Blastocystis* [39]. After getting into the intestinal lumen, the cysts first develop into the vacuolar form. In humans, vacuolar forms divide by binary fission and may develop into amoeboid, multivacuolar, or granular forms, before they become pre-cyst [9]. Vacuolar forms undergo encystation in the host intestine, while intermediate cyst forms may be surrounded by a thick fibrillar layer that is subsequently lost during passage into the external environment to infect other individuals. The pre-cyst may also develop into the thin-wall cyst and lead to autoinfection of the host [9]. Information about the transformation from the amoeboid to the vacuolar form and from the vacuolar to the cyst form is lacking. *Blastocystis* is a strict anaerobe and a common inhabitant of the human gastrointestinal tract, as well as other mammals. Many reports suggest that humans are potentially infected by five or more subtypes of *Blastocystis* [40], and that certain animals represent reservoirs for transmission to humans [1].

## Genetic diversity and pathogenicity of *Blastocystis* sp.

Based on SSU rDNA analysis, at least 17 different STs of *Blastocystis* were detected, which colonize a wide range of hosts, including humans and animals, both mammalian and non-mammalian. Some STs exhibit host specificity with variable geographic distribution. There is a high prevalence of ST1 and ST2 in America, ST1 and ST3 in Australia, Europe, and South Eastern Asia, and ST4 in Europe [8]. Humans are colonized mainly by ST1 through ST4, comprising over 90% of reports; however, depending on the regions and countries, infection by ST5 through ST9 is also observed [8, 41]. To date, ST10 through ST17 have not been found in humans [11, 41], with the exception of research by Ramírez et al. [42] in 2016, who reported, for the first time, human infection with ST12. Gender differences in the prevalence of *Blastocystis* STs have been reported. In Sweden, for instance, ST3 was found to be more common in males than in females, while in females, ST4 was almost as common as ST3. However, the difference in the relative frequency of ST4 between men and women was statistically insignificant [43].

In the recent literature, researchers have been debating over the correlation between distinct *Blastocystis* subtypes and their pathogenic potential. Clark was the first to suggest that different subtypes with different pathological potentials may exist [43]. With respect to that fact, in 2000, Kaneda et al. [27] suggested that STs 1, 2, and 4 might be responsible for gastrointestinal symptoms. In 2012, Poirier et al. [44] indicated that ST7 is correlated with IBS. Puthia et al. [45] have shown that, in rat epithelial cells, ST4 can induce apoptosis in a contact-independent manner to increase epithelial permeability. ST7 likely uses hydrolases to attack host tissues for its nutrient supply [44]. In 2006, Yan et al. [46] demonstrated only ST1 in a group of symptomatic patients, which was later confirmed by El Safadi et al. [47] in 2013, demonstrating that ST1 was associated with an elevated pathogenicity. The pathogenicity of ST4 was also hypothesized by Stensvold et al. [48] in a short report in 2011. The explanations for pathogenicity may include intra-subtype differences in *Blastocystis* protease activity, variations in intestinal microbiota of the individual host, and a symbiotic role for viruses associated with *Blastocystis*, which can interact to mediate host colonization and *Blastocystis* virulence [49, 50]. Also, the presence of gut microbiota seems to be essential for the pathogenic expression of enteric protozoan such as *Blastocystis*. The hypothesis of protozoa axenization by some bacteria has been proposed [51]. The cysteine proteases produced by ST4 and ST7 were revealed to be able to cleave human IgA in vitro [52, 53], as a mechanism for parasite survival and colonization in the gut, immune evasion, virulence, and cell cycle regulation [53]. Enzymes can also modulate inflammatory IL-8 production, as well as cathepsin B activation [54], and are able to increase the permeability of intestinal epithelial cells [45, 55].

In contrast, several studies showed no distinct differences in STs between isolates from symptomatic and asymptomatic groups of individuals with gastrointestinal disorders [39]. In addition to unspecific gastrointestinal symptoms, an association between the parasite subtypes and certain cutaneous disorders have been observed [56–59], such as the presence of *Blastocystis* ST2 [57], ST3 especially the amoeboid form, or ST4 in patients with acute or chronic urticaria (CU) [56, 58, 60–62]. Oxidative stress is probably is another reason why certain *Blastocystis* subtypes are more virulent. It is reported that *Blastocystis* infection correlates with a significant oxidative burst, leading to oxidative stress [63]. Some subtypes may induce higher concentrations of oxidative stress and precipitate skin reactions such as urticaria. A recent study showed that live *Blastocystis* parasites and whole cell lysate alone did not activate toll-like receptors in the human TLR reporter monocytic cell line, while live ST4-WR1 parasites inhibited the LPS-mediated NF-κB activation. In contrast, whole cell lysates of ST7-B and ST4-WR1 induced pleiotropic modulation of ligand-specific TLR-2 and TLR-4 activation, with a compounding effect of ST7-B on LPS-mediated NF-κB activation [63]. A higher caspase-like activity of *Blastocystis* spp. ST3 was found in isolates from individuals who had gastrointestinal symptoms [65].

## The human gut microbiota: current knowledge

The human microbiota is made up of different microbial communities present in different parts of the human body, such as the skin, vagina, oro-nasal cavity, esophagus, and gastrointestinal (GI) tract [66]. The human body is composed of about 10^13^ cells [67]. There are about ten times this number of microbial cells associated with the healthy human body [67]. These microbes interact with their host and have an impact on human health. The human GI microbiota is mostly concentrated in the colon and is made up of a majority of bacteria, a few archaea, known as the microbiome, viruses (the virome), fungi, and other uni- and multicellular eukaryotes, called protists and helminths, respectively [5, 68]. Only 30% of the human GI microbiota have been characterized [69]. It is difficult to describe the actual meaning of “normal” or “healthy” human GI microbiota. A “core microbiota” has been established [70], but it remains unclear what the essential constituents are. A number of studies have emphasized that the distribution of specific microbial communities among individuals could be influenced by several factors, including the geographical origin, age, diet of the studied individual, gender [71], stress, smoking, GI infections, as well as antibiotic or probiotic uptake [70, 72–78]. It is believed that the highly evolved biofilm communities that are closely associated with the intestinal epithelium are biologically more relevant than planktonic microbes that exist in the lumen of the gut or associated with food residue [79]. Furthermore, fecal specimens may not accurately portray the mucosal microbiota [72, 80]. And, also, there are significant differences between the microbiota of the liquid fraction compared with that associated with the solid phase [79].

The gut microbiota is typically dominated by bacteria and specifically by members of the divisions Bacteroidetes and Firmicutes [81]. From stomach to colon, the bacterial biomass ranges from 10^2–3^ to 10^11–12^ cells/mL [82]. Ninety-five percent of them are anaerobic bacteria and at least 1000 different species have been listed to date [83]. The most frequently detected in the human gut are the phyla of the Firmicutes, Bacteroidetes, Actinobacteria, Cyanobacteria, Fusobacteria, Proteobacteria, and Verrucomicrobia [84]. Knowledge concerning bacterial communities far outpaces that of the viral and eukaryotic communities [85]. The observed ratio of 7–10 viral-like particles per microbial cell in environmental [86] human samples [87] means that we could expect to find as many as 10^15^ phages in the body, with the gut having the highest abundance of viruses, 3 × 10^12^ [88]. The diversity of the human virome is low. It is estimated that there are 1500 viral genotypes in a typical healthy, human virome [88]. Studies of the viral community in the human gut showed Caudovirales to be prevalent [89]: Siphoviridae, Myoviridae, Podoviridae, Microviridae [90, 91]. Eukaryotes that reside in the human gut are distributed across the eukaryotic tree and their relationship with the human host varies from parasitic to opportunistic to commensal to mutualistic [92]. It has been known that yeasts constitute part of the intestinal flora: *Candida* spp. (*C. parapsilosis*, *C. albicans*, *C. glabrata*, *C. krusei*, *C. tropicalis*) or *Saccharomyces boulardii* [92, 93]. Diverse eukaryotes inhabit the human gut, including protists and helminths [92]. Many species of protists are commensals and harmless, including *Pentatrichomonas* and *Entamoeba dispar* [94]. Interestingly, available data suggest that many common eukaryotic residents are commensals, but are, in some cases, associated with gastrointestinal disorders [5]. Good examples are *Blastocystis* sp. or *Dientamoeba fragilis* [5, 95]. Other representative protists that are part of the human gut eukaryome include: *Enteromonas hominis*, *Retortamonas intestinalis*, *Chilomastix mesnili*, *Entamoeba hartmanni*, *Entamoeba nana*, *Entamoeba coli*, *Isospora belli*, *Iodamoeba buetschi* and *Encephalitozoon cuniculi* [5]. Pathogenicity is certainly the role for some intestinal protists, such as *Cryptosporidium* spp., *Entamoeba histolytica* or *Giardia intestinalis* [92], as well as for some helminths, such as *Ascaris lumbricoides* and *Strongyloides stercoralis* [5].

## The role of *Blastocystis* in the human gut: bad and good sites

*Blastocystis* was first described more than 100 years ago, but there are still many obscurities about its clinical significance. It may colonize the bowels and is commonly isolated in individuals with neither gastrointestinal complaints nor symptoms [19, 47]. However, *Blastocystis* was also identified as the only causative agent of gastrointestinal or dermatological symptoms, such as abdominal pain, diarrhea, nausea, vomiting, bloating, anorexia, or, less commonly, urticaria, intense itching [56, 57], or iron deficiency anemia [96–98].

As mentioned above, there can be several explanations for *Blastocystis* pathogenicity. It may depend on the *Blastocystis* subtype: different subtypes can excrete protease enzymes which are able to take part in the induction of virulence [49, 99, 100]. Most *Blastocystis* isolates found in stool samples are in cyst or vacuolar forms. The amoeboid form is rarely seen, but is mostly associated with symptoms [3]. Therefore, amoeboid forms of *Blastocystis* are probably pathogenic. A large number of the parasites in the intestine (>5 parasites per high-power field) are also connected with gastrointestinal symptoms [1]. Human gut microbiota composition and the immune system are also deciding agents in the pathogenicity and occurrence of the parasite. In immunocompromised individuals, such as those with HIV infection, the prevalence reaches 38% in developed countries [28], and an association with diarrhea is suggested. The nutritional status of an individual may be one of the most important risk factors for co-infection [3]. Children and the elderly are highly susceptible to *Blastocystis* infection [20, 31], whereas research interestingly showed that people aged 30–50 years old are mostly infected [33]. *Blastocystis* can also have beneficial effects stemming from its ability to modulate the host immune system. One mechanism of action is the stimulation of mucus production, via the cytokine IL-22, which alleviates symptoms of colitis, improving gut health [101]. Perhaps *Blastocystis* is more common in healthy people because it helps maintain a healthy mucus layer in the intestine, either directly or through interactions with beneficial bacteria or the immune system [5]. *Blastocystis* have been shown to be more prevalent in patients suffering from IBS [18, 102–106]. A 2014 study by Nourrisson et al. [107] suggests that *Blastocystis* may be used as an indicator of microbiota changes; a lower abundance of *Bifidobacterium* spp. and *Faecalibacterium prausnitzii* were reported to have protective and anti-inflammatory effects, which could lead to intestinal dysbiosis and IBS. Nagel et al. [108] confirmed these results in 2016. On the other hand, in 2016, Audebert et al. [109] suggested that colonization by *Blastocystis* could be associated with a healthy gut microbiota. Their study showed a higher bacterial diversity in *Blastocystis*-colonized patients compared to that identified in *Blastocystis*-free individuals. In *Blastocystis*-colonized patients, there was a higher abundance of the Clostridia class and Ruminococcaceae and Prevotellaceae families, while Enterobacteriaceae were enriched in *Blastocystis*-free patients. The most recent results of the latest studies leave the pathogenicity of *Blastocystis* still unclear and this is similar to the chicken and egg question: which came first? It is still a mystery. Is *Blastocystis* an agent of the gut dysbiosis and changing the microbiotic diversity, or are the metabolic dysfunctions and changes in the content of microbiota the reason for the higher colonization by *Blastocystis*? There is a possibility that some species of bacteria are triggering the protease activity of *Blastocystis*, which causes the gastrointestinal symptoms. It may also depend on parasitic subtype. To address this, further studies in humans are required [109].

## The influence of different diets on *Blastocystis* as compared to antibiotic treatment

Although many *Blastocystis* infections remain asymptomatic, recent data suggest that it is a frequent cause of gastrointestinal symptoms in children and adults [110]. Many parasitologists insist that, when *Blastocystis* organisms are present in large numbers in stool examination, even without the presence of any other known bacterial, viral, or parasitic infection, treatment should be proposed [110, 111]. Therapy should be limited to patients with relentless symptoms and a complete negative workup for alternative etiologies. Several drugs have been used against *Blastocystis* infection, the most common still being metronidazole (MTZ), as the first-line treatment, followed by nitazoxanide (NTZ), trimethoprim–sulfamethoxazole (TMP-SMX), ketoconazole, and tinidazole as secondary treatments. Studies have shown that, while MTZ demonstrates effectiveness in some individuals [112, 113], it has also been shown to exhibit side effects and resistance in others [114, 115]. Most probably, it depends on the *Blastocystis* subtypes. Girish et al. [116], in 2015, reported that STs 1, 3, and 5 are susceptible to MTZ, but resistant to ketoconazole, even when high doses were administrated. ST1 is also resistant to TMP-SMX in lower doses, and ST3 to NTZ in lower doses [116]. Studies have shown STs 4 and 7 to be resistant to MTZ [117]. This drug can cause undesirable side effects and changes in the gut microbiota. Moreover, failures in treatment are frequently reported [117–122]. Additionally, potential carcinogenic, teratogenic, and embryotoxic effects of metronidazole have been reported [123]. Therefore, there is a need to develop safe and alternative antimicrobial agents, such as the use of medicinal plants, spices, specific vegetables, or yeasts.

The number of trials, in vitro and in vivo, in which the anti-*Blastocystis* efficacies of some local plants are assessed have been on the rise lately [124]. Plants were chosen for their antimicrobial activity and chemical composition. Garlic (*Allium sativum*) contains a wide range of the thiosulfinates (e.g., allicin), which are responsible for the antibacterial activity [125] related to the inhibition of enzymes, including thiol in microorganisms [126, 127]. Moreover, allicin in the garlic acts by totally inhibiting RNA synthesis and partially inhibiting DNA and protein synthesis of the parasites [128]. Additionally, hexahydrocurcumin, a constituent isolated from ginger (*Zingiber officinale*), might be effective in killing the parasites [129]. Yakoob et al. [130] in 2011 and Abdel-Hafeez et al. [131] in 2015 proved activity against *Blastocystis* with garlic extract in vitro as compared to the antimicrobial drugs MTZ and NTZ, respectively. Interestingly, they also tested the antiparasitic activity of ginger and had differing results. As Yakoobs and colleagues [130] showed in their study, *Blastocystis* STs 1 and 3 were not sensitive to ginger, black pepper, or cumin when compared to garlic and MTZ. According to the study of Abdel-Hafeez et al. [131], ginger has the greatest effect on *Blastocystis* clinical isolates, but onion (*Allium cepa*) or turmeric (*Curcuma longa*) do not [131, 132]. Moreover, garlic and ginger decrease malondialdehyde (MDA) production significantly. The allicin contained in garlic acts as an antioxidant by retrieving reactive oxygen species (ROS), preventing lipid oxidation and production of pro-inflammatory messengers [133]. Additionally, gingerol contained in ginger inhibits the ascorbate/ferrous complex-induced lipid peroxidation [134]. Dugasani et al. [135], in 2010, reported that gingerols and shogaols are the most bioactive compounds of ginger. Both garlic and ginger seem to be strong antioxidants inhibiting nitric oxide (NO) production [131, 135]. Intestinal NO increases upon *Blastocystis* infection as a host defense mechanism of epithelial cells against parasites [117, 131]. Mirza et al. [117], in 2011, suggested that *Blastocystis* is susceptible to NO. Nitric oxide is important in homeostasis and host defense; however, it may also lead to cellular damage and gut barrier failure, as well as having been involved in the pathogenesis of many inflammatory and autoimmune diseases [136, 137]. Garlic and ginger treatments significantly downregulated NO intestinal release [131]. This may be caused by a decrease in *Blastocystis* loads in the intestine. Downregulation of MDA and NO are important mechanisms of garlic- and ginger-induced antiparasitic effects.

El Deeb et al. [138], in 2012, proved an inhibitory effect of *Ferula asafoetida* (in both powder and oil form) on *Blastocystis* ST3. Furthermore, asafetida exerted a detrimental effect on *Blastocystis* morphology, which was especially obvious at higher concentrations. The viable vacuolar forms which were typically seen before incubation with either powder or oil were replaced by more granular forms, which lost viability over time and showed a shriveled appearance [138]. Asafoetida consists principally of volatile oil (4–20%) with isobutyl propanyl disulfide (C8H16S2), resin (40–60%) with ester of asaresinotannol and free ferulic acid, and gum (∼25%) with caffeic acid cinnamyl ester showing moderate activity for inhibiting LPS-induced nitric oxide production in murine macrophage RAW264.7 cells [139]. *Blastocystis* ST3 is also sensitive to Tongkat Ali (*Eurycoma longifolia*), as Girish and colleagues proved in a study in 2015 [116]. Most likely, four compounds are responsible for antiparasitic activity: 3,4-dihydrochaparrinone, laurycolactone B, β-carboline-1-propionic acid, and canthin-6-one. These compounds have previously been proven to possess therapeutic properties [140, 141]. Recently, there have been many reports which have shown the inhibitory effect of herbal extracts and spices on *Blastocystis*. Vital and Rivera [142], in 2009, proved that ethanol extracts of leaves of *Chromolaena odorata* and ethyl acetate extracts of stem bark of *Uncaria perrottetii* inhibited *Blastocystis* growth and decreased cell counts at 0.5 and 1.0% concentrations, respectively. Furthermore, Özbílgín et al. [143], in a 2013 study, demonstrated that the methanol extract of *Achillea millefolium* gave promising results and could be used as an anti-protozoal agent in the future, especially against *Blastocystis* STs 1, 2, and 3. Also, El Wakil [144], in 2007, showed that an aqueous extract of *Nigella sativa* significantly inhibits the growth of *Blastocystis* isolates. In addition, some medicinal plants from Ghana and Thailand showed high activity against *Blastocystis* [145, 146]. Similarly, supplementation with 600 mg emulsified oil of Mediterranean oregano (*Origanum vulgare*) daily lead to the complete disappearance of *Blastocystis* [147]. It is also suggested to use *Saccharomyces boulardii* to cure *Blastocystis* infection [111]. Commensal yeasts act as a regulator of homeostasis in the gut through preventing the colonization of pathogenic agents on intestinal mucosa and augmenting the local immune response [148]. In a placebo-controlled study, it was found to be more effective against *Blastocystis* when compared to metronidazole [111].

## Conclusions

*Blastocystis* sp. is a parasite which does not need to be cured with the antibiotics that cause side effects, such as metronidazole (MTZ). Mainly, the choice of eradication depends on the *Blastocystis* subtype, geographic region of occurrence, pathogenicity, immune system of the host, human gut microbiota, or chronic diseases, such as diabetes. Natural herbs, vegetables, or spices as an alternative for blastocystosis treatment not only reduce drug resistance, but also their side effects and the cost of treatment, especially in developing countries. Special diets are effective mainly by inhibiting parasitic enzyme activity, RNA, DNA, and protein synthesis, and, also, nitric oxide (NO) production. Moreover, commensal yeasts and bacteria prevent the colonization of pathogenic agents on intestinal mucosa and augment the local immune response. Certain species of beneficial microorganisms can have a negative influence on parasites by producing molecules that trigger the immune system, but they may also cause protease activity in *Blastocystis*, leading to symptoms of infection. Further investigation needs to be done to identify the organic compounds causing *Blastocystis* eradication. Research should address if the treatment directly affects *Blastocystis* or if it acts by destroying the bacterial flora necessary for its development, or both. We would like to suggest for future research the determination of whether *Blastocystis* is an agent changing the human gut microbiota or the opposite; does the commensal microbiota help the parasite to colonize the gastrointestinal tract? These questions and many others still remain unclear.
